# Influence of milling pH and storage on quality characteristics, mineral and fatty acid profile of buffalo Mozzarella cheese

**DOI:** 10.1186/s12944-019-0976-9

**Published:** 2019-01-29

**Authors:** Nabila Gulzar, Saima Rafiq, Muhammad Nadeem, Muhammad Imran, Anjum Khalique, Iqra Muqada Sleem, Tahir Saleem

**Affiliations:** 1 Department of Dairy Technology, University of Veterinary and Animal Sciences, Lahore, 55300 Pakistan; 2 Department of Food Technology, University of Poonch, Rawalakot, 12350 Pakistan; 3 Institute of Home and Food Sciences, Faculty of Life Sciences, Government College University, Faisalabad, 38000 Pakistan

**Keywords:** Mozzarella, Fatty acid profile, Milling, pH, Storage

## Abstract

**Background:**

Currently, cheese fat is a major component of human diet due to change in eating habits. It contains a number of health destroying as well as health promoting fatty acids. Bovine milk cheese fatty acid composition is regulating by many factors. These may be breed of animal, animal health condition like mastitis and stage of lactation. It also differs with feed and dietary fat intake and seasons. Many studies demonstrated physicochemical, textural and sensory characteristics of Mozzarella cheese with variation in technological process but no literature found about the fatty acid profile and potential influence of milling pH on the fatty acid composition of buffalo Mozzarella cheese.

**Methods:**

Buffalo Mozzarella cheeses were manufactured at 5.2, 5.1, 5.0, 4.9 and 4.8 milling pH, vacuum packaged and stored at 4 °C and analyzed for quality characteristics, mineral composition and fatty acid profile on days 1, 45, and 90. Results were analyzed by ANOVA according to complete randomized design.

**Results:**

This study evaluated the effect of milling pH on chemical composition, mineral and fatty acid profile of buffalo Mozzarella cheese. Experimentally induced milling pH differences persisted and significantly affected chemical composition during first day of manufacturing but have no effect on fatty acid profile of cheese. However, storage effects significantly on chemical composition and fatty acid profile of cheese. Decreasing milling pH from 5.2–4.9 resulted in decrease in moisture content of cheese. As a result of changes in milling pH, all the cheeses experienced a significant loss in protein content. In contrast to protein content, fat content of cheese increases with decreasing milling pH. Ash contents of cheese decreased with decreasing milling pH. The level of calcium decreases from 77.82 mg/g to 69.1 mg/g with decreasing milling pH while there is no clear trend observed for potassium and sodium during change in milling pH. Saturated fatty acids presented higher concentrations reaching values of about 71.38 g/100 g throughout storage while monounsaturated fatty acids decreases with storage from 26.72 to 22.06 g/100 g. On the other hand, total polyunsaturated fatty acids exhibited lower concentrations than total monounsaturated fatty acids reaching values of 3.2 g/100 g and its value also decreased with ripening and reached to 1.6 g/100 g. Concentration of C18:1 *t*10–11 was observed 1.89% in freshly prepared cheese. Milling pH did not influence C18:1 *t*10–11 concentration but storage days significantly (*p* < 0.05) decreased its concentration.

**Conclusion:**

In modern era, Mozzarella cheese is major source of dietary fatty acids. The study demonstrated that Mozzarella cheese is a rich source of saturated fatty acids that has detrimental effect on health but it is also observed that it is also a major source of essential fatty acids that has beneficial impact on health. It is concluded that technological conditions like milling pH minimally influence cheese fatty acid profile but after manufacturing treatments and conditions like packaging and storage greatly influence fatty acid profile of cheese. It was concluded that cheese may get oxidized if it is packed in inappropriate packaging material that have reduced air barrier resistance. Moreover, cheese storage under light may also become oxidized which is also harmful for health.

## Background

Worldwide, consumer’s attention has been started to be focused on foods that have well specified health impacts. One of the important component of food that concerns more to consumer’s health is the fatty acid profile of food which includes short, intermediate and long chain fatty acids [1]. Recent advices for a good diet however, recommends less consumption of dairy fat, because of its saturated fatty acids, which have hypercholesterolemic effect. But dairy fat is also important due to presence of unsaturated fatty acids which is helpful in reducing blood cholesterol content and also provide protection against cancer and cardiovascular diseases [2].Changing lifestyle, eating habits and use of innovative technology in processing of Mozzarella cheese demands tailor made characteristics of this cheese. Therefore, cheese manufacturer are producing cheese that is well suiting to consumers requirements. In this aspect, increase in interest of consumers to know the fatty acid (FA) composition of cheese is continuously increasing, as certain unsaturated FA are recognized as bioactive substances [3]. Mozzarella cheese has become one of the dominant cheese varieties all over the world due to its primary utilization in pizza preparation. Buffalo milk is most demanded for Mozzarella due to its high fat and presence of essential fatty acids [4]. Pakistan is the 5th largest milk producer of the world and ‘Black Gold of Asia’ commonly known as buffalo milk donate a major part in total milk production of Pakistan and this black gold is very suitable for the production of mozzarella cheese [5].

Dairy fat is a vital component of human diet owing to their essential functions perform in to the body. Milk fat triacylglycerol are created from more than 400 types of fatty [6].The saturated and unsaturated fatty acids in milk accounts for about 70 and 27% by weight [7]. While, 2.7% of the fatty acids occur in Trans form. Many factors are associated with the variation in the amount and fatty acid composition of bovine milk lipids [8]. They may be of animal origin, i.e. related to genetics (breed and selection), stage of lactation, mastitis and ruminal fermentation, or they may be feed-related factors, i.e. related to fiber and energy intake, dietary fats, and seasonal and regional effects. In most of countries many customers are happy to pay more for handmade cheese as compared to cheese made on industrial scale. As there is a general adverse nutritional image that handmade cheese has attractive flavor and texture. But with a rising number of customers who are more careful about their food selections, artisanal cheese can’t be surpassed. Although milling pH processing step exist in both artisanal and industrial processing but artisanal producers get benefit of milk quality that differs with season and feed of animal. The animal feed affect the quality of cheese as different grazing pastures gives different fatty acid profile and flavor of cheese [9]. Artisanal cheesemakers change manufacturing schedule of different cheese varieties or change processing steps with time of year. It is concluded that artisanal cheese quality not solely depends on method of manufacturing but other parameters also influence cheese quality. Various researches indicating milk processing conditions that affect fatty acid profile of developed product. As the effects of processing conditions like heating, cooking, storage and aging on the fatty acid profile of milk fat and dairy products have often been deliberated disputed. Ha et al. [10] stated higher Conjugate Linoleic Acid (CLA) contents in cheese fat as compared to milk fat. Some scientist observed no change on the fatty acid composition of cheeses including Pecorino Romano cheese, blue cheese, Swiss cheese, Emmental cheese and Ricotta cheese [11, 12].In one study it is stated that processing, such as heating, can change the CLA isomer distribution in dairy products while the total CLA content is unchanged by conventional processing [13]. Lin et al. [14] observed significantly lower CLA content in vacuum packed cheese as compared to canned cheese. They also found minor influence of milling pH and antioxidant on fatty acid composition of Cheddar cheese. Gürsoy et al. [15] examined 30 commercial Turkish hard and soft cheeses and detected higher CLA content in aged hard cheeses. Some conditions other than processing may also influence fatty acid profile of cheese like diet of animal and season. Santillo et al. [16] studied the influence of dietary supplementation of flax seed on the fatty acid profile of milk and Cacioricotta cheese from Italian Simmental cows and concluded that long chain fatty acids particularly linolenic acid was found to be higher in cheese made from milk of cows fed with flaxseed. Seasonal variation also influence the fatty acid profile of cheese as it is directly or indirectly related to the diet of the animal. Pasture quality varies with the change in season [9].These contradictory reports about the fatty acid profile of cheeses do not countenance decisive remarks to be drawn. Modern dietary guidelines recommend knowing the fatty acid profile of each food item before consuming. Mozzarella cheese milling pH (The pH at which cheese is milled into small pieces and is ready for pasta filata process) is an important step that affect all functional and sensory characteristics of cheese. Formation of curd chips depends on milling pH which ultimately influence cheese composition and functional characteristics. Guinee et al. [17] studied that curd disintegrate at 5.75 milling pH. They found that curd will form its proper texture at 5.3 milling pH in case of cheddar cheese. Gulzar et al. [18] found that buffalo Mozzarella cheese exhibit more stretch properties at 4.9 milling pH. At higher milling pH curd do not stretch and disintegrate in hot water during pasta filata process and loses of fat occurs. Although many reports about influence of milling pH on physicochemical and functional characteristics of Mozzarella cheese have been given by several workers but, little literature found about the fatty acids profile and potential influence of milling pH on the fatty acid composition of buffalo Mozzarella cheese. In this study, we thus concentrated on the fatty acid composition of buffalo Mozzarella cheese at various milling pH.

## Materials and methods

Heat stretched semi-soft “Mozzarella” cheese samples was made using raw milk exclusively from buffalo milk procured from Dairy Animals Training and Research Center University of Veterinary and Animal Sciences, Lahore. For cheese making, partially skimmed (0.9 casein to fat ratio) pasteurized buffalo milk was inoculated and coagulated with thermophilic starter at 37 °C and bovine rennet respectively. Starter culture and rennet take 1 h and 50 min to develop curd and pH of 6.4. The curd is cut with knife into 3–4 mm curd particles. The curd-whey mixture is heated to 37–42 °C till 6.2 pH for whey removal. This curd-whey heating process continue for 30 min. The curd is placed for cheddaring process until 5.2, 5.1, 5.0, 4.9 and 4.8 milling pH resulted five treatment samples (M5.2, M5.1, M5.0, M4.9 and M 4.8). Cheddaring process last for 1 h and 10 min to develop required pH. The milled cheese was heat stretched at 94 °C and vacuum packed for a storage period of 90 days in polyethylene nylon food vacuum plastic bags of 100 μm thickness.

### Physicochemical analysis

Moisture, Acidity, Ash [19], pH [20], fat [21] and protein [22] determinations in sample cheeses were performed according to prescribed methods.

### Minerals analysis

Sodium potassium and calcium contents were determined using flame photometer as described by [23].

### Fatty acid analysis

The fatty acid methyl esters were prepared by trans-esterification with potassium hydroxide according to method of ISO 5509:2000E [24]. The injected volume was 1 μL. Each sample was analyzed three times. Fatty acid profile of Mozzarella cheese including CLA was determined on GC-MS (Agilent Technologies, Model 7890-B) using SP-2560 fused silica capillary column (60 m long, 0.25 mm internal diameter, 0.25um phase thickness) on a flame ionization detector. Injection volume was 1 1 μL, the temperature program and operating conditions were as: injector and detector temperature 200 and 250 °C; carrier gas helium at 1.2 ml/min, hydrogen 4 ml/min and oxygen 40 ml/min, in a split mode, the column was maintained at 50 °C for 1 min then ramped at 10 °C/min to 220 °C and finally 3 min at 250 °C. Fatty acids were identified and quantified by FAME-37 standard (Sigma Aldrich, USA).

### Sensory analysis

Sensory characteristics of Mozzarella cheese were determined in a well ventilated and illuminated sensory evaluation laboratory. Panel of ten trained judges were asked to adjudge color, appearance, texture) by the method [25].

### Statistical analysis

Every treatment was run in triplicate, experiment was planned in a Completely Randomized Design (CRD) and data were analyzed by two way analysis of variance and significant difference among the treatments was determined by Duncan Multiple Range Test using SAS 9.1 software [26].

## Results and discussions

### Physicochemical analysis of buffalo Milk and cheese

The means of the physicochemical parameters (fat, protein, pH, acidity, total solids and calcium content) in buffalo milk were found (6.65, 4.5%, 6.6, 0.12, 12.12% and 1.7 mg/g) respectively. While, the physicochemical analysis of cheeses are given in Table 1.

**Table 1:** Mean and standard error of physicochemical composition of buffalo milk mozzarella cheese at different milling pH during storage

Treatments	Storage Days	pH	Acidity (%)	Protein (%)	Fat (%)	Ash (%)	Moisture (%)
M4.9 (Control)	1st day	4.90 ± 0.00^a^	0.92 ± 0.01^c^	25.26 ± 0.15^d^	22.00 ± 0.05^a^	2.33 ± 0.01^d^	46.23 ± 0.09^a^
	45 days	4.86 ± 0.01^b^	1.51 ± 0.01^b^	25.16 ± 0.03^d^	22.46 ± 0.09^a^	2.32 ± 0.01^d^	45.23 ± 0.09^b^
	90 days	4.62 ± 0.01^c^	2.32 ± 0.01^a^	25.1 ± 0.02^d^	22.03 ± 0.03^a^	2.33 ± 0.01^d^	44.23 ± 0.09^c^
M4.8	1st day	4.8 ± 0.00^a^	1.08 ± 0.04^a^	24.1 ± 0.06^d^	22.15 ± 0.00^a^	2.16 ± 0.03^e^	45.13 ± 0.09^a^
	45 days	4.73 ± 0.02^b^	1.92 ± 0.01^b^	24.13 ± 0.03^d^	22.06 ± 0.03^a^	2.18 ± 0.04^e^	43.13 ± 0.01^b^
	90 days	4.51 ± 0.01^c^	2.52 ± 0.01^c^	24.04 ± 0.03^d^	22.16 ± 0.09^a^	2.18 ± 0.04^e^	42.13 ± 0.09^c^
M5.0	1st day	5.0 ± 0.00^a^	0.83 ± 0.01^c^	25.53 ± 0.15^c^	20 ± 0.00^b^	2.45 ± 0.00^c^	47.11 ± 0.06^a^
	45 days	4.95 ± 0.01^b^	0.93 ± 0.01^b^	25.81 ± 0.01^c^	20.2 ± 0.06^b^	2.45 ± 0.01^c^	46.11 ± 0.06^b^
	90 days	4.76 ± 0.02^c^	1.13 ± 0.02^a^	25.1 ± 0.06^c^	20.03 ± 0.03^b^	2.45 ± 0.00^c^	45.11 ± 0.06^c^
M5.1	1st day	5.1 ± 0.01^a^	0.74 ± 0.02^c^	26.33 ± 0.03^b^	19.0 ± 0.01^c^	2.74 ± 0.05^b^	52.3 ± 0.07^a^
	45 days	4.99 ± 0.01^b^	0.82 ± 0.02^b^	26.52 ± 0.01^b^	19.1 ± 0.02^c^	2.73 ± 0.03^b^	50.3 ± 0.06^b^
	90 days	4.90 ± 0.03^c^	0.87 ± 0.01^a^	26.41 ± 0.02^b^	19.16 ± 0.03^c^	2.73 ± 0.03^b^	49.8 ± 0.05^c^
M5.2	1st day	5.20 ± 0.00^a^	0.62 ± 0.01^c^	27.72 ± 0.02^a^	17.0 ± 0.03^d^	2.91 ± 0.02^a^	53.79 ± 0.06^a^
	45 days	4.88 ± 0.03^b^	0.92 ± 0.02^b^	26.3 ± 0.03^b^	19.23 ± 0.02^c^	2.74 ± 0.03^b^	50.36 ± 0.07^b^
	90 days	4.81 ± 0.01^c^	0.82 ± 0.02^a^	26.86 ± 0.04^a^	17.3 ± 0.01^d^	2.92 ± 0.02^a^	49.79 ± 0.06^c^

Values are expressed as mean ± SD of three replicates

^a,b,c,d,e^ different short case letters within column showed significant (*P* < 0.05) relationship

M4.9 (Cheese heat stretched at milling pH 4.9), M 4.8 (Cheese heat stretched at milling pH 4.8), M5.0 (Cheese heat stretched at milling pH 5.0), M5.1 (Cheese was heat stretched at milling pH 5.1), M5.2 (Cheese was heat stretched at milling pH 4.9)

### Mineral analysis

The changes in levels of calcium, potassium and sodium in cheese made with milling pH of 5.2, 5.1, 5.0, 4.9 and 4.8 during 90 days of storage are shown in Table 2.

**Table 2:** Mean and standard error of mineral composition of buffalo milk mozzarella cheese at different milling pH during storage

Treatments	Storage Days	Calcium (mg/g)	Potassium (mg/g)	Sodium (mg/g)
M4.9 (Control)	1st day	72.16 ± 0.60^e^	8.60 ± 0.87^a^	12.33 ± 0.43^d^
	45 days	72.19 ± 0.60^e^	8.26 ± 0.56^a^	12.60 ± 0.32^d^
	90 days	72.15 ± 0.50^e^	8.73 ± 0.67^a^	12.66 ± 0.54^d^
M4.8	1st day	69.10 ± 0.60^f^	8.23 ± 0.45^a^	12.13 ± 0.21^d^
	45 days	68.76 ± 0.50^f^	8.56 ± 0.65^a^	12.16 ± 0.61^d^
	90 days	68.76 ± 0.30^f^	8.73 ± 0.76^a^	12.13 ± 0.63^d^
M5.0	1st day	74.23 ± 0.33^c^	8.96 ± 0.94^a^	12.96 ± 0.78^b^
	45 days	73.9 ± 0.44^d^	8.63 ± 0.45^a^	12.96 ± 0.91^b^
	90 days	73.9 ± 0.33^d^	8.40 ± 0.91^a^	13.16 ± 0.43^b^
M5.1	1st day	75.26 ± 0.54^b^	8.30 ± 0.76^a^	12.76 ± 0.78^c^
	45 days	75.4 ± 0.56^b^	8.46 ± 0.58^a^	12.76 ± 0.76^c^
	90 days	75.4 ± 0.67^b^	8.48 ± 0.72^a^	12.8 ± 0.12^c^
M5.2	1st day	79.03 ± 0.34^a^	8.10 ± 0.56^a^	13.1 ± 0.45^b^
	45 days	75.4 ± 0.45^b^	8.50 ± 0.43^a^	12.83 ± 0.78^a^
	90 days	79.03 ± 0.65^a^	8.63 ± 0.43^a^	13.23 ± 0.98^b^

Values are expressed as mean ± SD of three replicates

^a,b,c,d,e,f^different short case letters within column showed significant (*P* < 0.05) relationship

M4.9 (Cheese heat stretched at milling pH 4.9), M 4.8 (Cheese heat stretched at milling pH 4.8), M5.0 (Cheese heat stretched at milling pH 5.0), M5.1 (Cheese was heat stretched at milling pH 5.1), M5.2 (Cheese was heat stretched at milling pH 4.9)

### Fatty acid profile of cheese

Statistical results indicated that milling pH effects non-significantly while ripening affects significantly on fatty acid profile of Mozzarella cheese. Table 3 showed the saturated (SFA), monounsaturated (MUFA) and polyunsaturated (PUFA) fatty acid profiles of Mozzarella cheese prepared at various milling pH. SFA presented higher concentrations than total MUFA reaching values of 71.38 and 26.7 g/100 g lipids (Table 3), respectively, at 1st day. On the other hand, total PUFA exhibited lower concentrations than total MUFA reaching values of 3.2 g/100 g lipids at first day (Table 3). Saturated fatty acids presented higher concentrations reaching values of about 71.38 g/100 g throughout storage while monounsaturated fatty acids decreases with storage from 26.72 to 22.06 g/100 g. On the other hand, total polyunsaturated fatty acids exhibited lower concentrations than total monounsaturated fatty acids reaching values of 3.2 g/100 g and its value also decreased with ripening and reached to 1.6 g/100 g. The amount of CLA (C18:1 *t*10–11) was found 1.89% in freshly prepared cheese. Milling pH has no influence C18:1 *t*10–11 concentration but storage period significantly (*P* < 0.05) decreased its concentration.

**Table 3:** Mean and standard error of fatty acid profile of buffalo milk mozzarella cheese at different milling pH during storage

Fatty acid	Storage Days	M4.9 (Control)	M4.8	M5.0	M5.1	M5.2
Butyric acid C4:0	1st Day	1.86 ± 0.21^a^	1.86 ± 0.24^a^	1.85 ± 0.31^a^	1.86 ± 0.41^a^	1.84 ± 0.21^a^
	45 Days	1.84 ± 0.41^a^	1.85 ± 0.33^a^	1.86 ± 0.22^a^	1.85 ± 0.31^a^	1.83 ± 0.41^a^
	90 Days	1.84 ± 0.31^a^	1.82 ± 0.24^a^	1.83 ± 0.26^a^	1.83 ± 0.45^a^	1.82 ± 0.32^a^
Caproic acid	1st Day	1.66 ± 0.26^a^	1.63 ± 0.26^a^	1.63 ± 0.21^a^	1.64 ± 0.25^a^	1.65 ± 0.41^a^
	45 Days	1.65 ± 0.61^a^	1.66 ± 0.51^a^	1.65 ± 0.26^a^	1.64 ± 0.46^a^	1.63 ± 0.26^a^
	90 Days	1.66 ± 0.45^a^	1.63 ± 0.56^a^	1.65 ± 0.52^a^	1.66 ± 0.24^a^	1.66 ± 0.17^a^
Caprylic acid	1st Day	1.03 ± 0.22^a^	1.02 ± 0.29^a^	1.01 ± 0.11^a^	1.03 ± 0.32^a^	1.02 ± 0.31^a^
	45 Days	1.01 ± 0.21^a^	1.03 ± 0.24^a^	1.04 ± 0.23^a^	1.03 ± 0.22^a^	1.03 ± 0.22^a^
	90 Days	1.05 ± 0.29^a^	1.03 ± 0.31^a^	1.03 ± 0.35^a^	1.03 ± 0.25^a^	1.03 ± 0.21^a^
Capric acid	1st Day	2.98 ± 0.43^a^	2.96 ± 0.46^a^	2.96 ± 0.31^a^	2.97 ± 0.47^a^	2.98 ± 0.39^a^
	45 Days	2.95 ± 0.24^a^	2.95 ± 0.15^a^	2.94 ± 0.22^a^	2.93 ± 0.34^a^	2.91 ± 0.51^a^
	90 Days	2.96 ± 0.61^a^	2.94 ± 0.52^a^	2.93 ± 0.41^a^	2.93 ± 0.53^a^	2.94 ± 0.41^a^
Lauric acid	1st Day	2.80 ± 0.24^b^	2.70 ± 0.24^b^	2.70 ± 0.21^b^	2.81 ± 0.21^b^	2.82 ± 0.21^b^
	45 Days	2.81 ± 0.11^a^	2.83 ± 0.15^a^	2.84 ± 0.21^a^	2.81 ± 0.35^a^	2.81 ± 0.45^a^
	90 Days	2.83 ± 0.99^a^	2.80 ± 0.60^a^	2.82 ± 0.88^a^	2.81 ± 0.65^a^	2.82 ± 0.41^a^
Myristic acid	1st Day	15.16 ± 0.25^b^	15.14 ± 0.41^b^	15.14 ± 0.33^b^	15.16 ± 0.24^b^	15.15 ± 0.25^b^
	45 Days	15.76 ± 0.22^b^	15.75 ± 0.43^a^	15.74 ± 0.22^a^	15.72 ± 0.25^a^	15.71 ± 0.53^a^
	90 Days	15.6 ± 0.27^a^	15.56 ± 0.43^a^	15.54 ± 0.43^a^	15.40 ± 0.23^a^	15.54 ± 0.11^a^
Palmitic acid	1st Day	25.52 ± 0.42^a^	25.43 ± 0.13^a^	25.56 ± 0.52^a^	25.51 ± 0.51^a^	25.52 ± 0.21^a^
	45 Days	25.34 ± 0.22^a^	25.33 ± 0.26^a^	25.33 ± 0.29^a^	25.21 ± 0.24^a^	25.12 ± 0.23^a^
	90 Days	25.23 ± 0.26^a^	25.23 ± 0.46^a^	25.23 ± 0.47^a^	25.23 ± 0.61^a^	25.23 ± 0.43^a^
Palmitoleic acid	1st Day	1.026 ± 0.25^a^	1.1 ± 0.25^a^	1.36 ± 0.25^a^	1.01 ± 0.25^a^	1.16 ± 0.41^a^
	45 Days	0.901b ± 0.24^b^	0.92b ± 0.25^b^	0.95b ± 0.23^b^	0.99b ± 0.14^b^	0.9b ± 0.59^b^
	90 Days	0.85c ± 0.43^c^	0.82c ± 0.33^c^	0.80c ± 0.41^c^	0.82c ± 0.54^c^	0.80c ± 0.43^c^
Stearic acid	1st Day	17.97 ± 0.43^a^	17.93 ± 0.22^a^	17.93 ± 0.55^a^	17.91 ± 0.66^a^	17.95 ± 0.24^a^
	45 Days	17.95 ± 0.43^a^	17.94 ± 0.57^a^	17.94 ± 0.51^a^	17.99 ± 0.23^a^	17.91 ± 0.43^a^
	90 Days	17.25 ± 0.64^b^	17.23 ± 0.45^b^	17.20 ± 0.9^b^	17.19 ± 0.98^b^	17.18 ± 0.37^b^
Oleic acid	1st Day	25.09 ± 0.49^a^	25.01 ± 0.51^a^	24.09 ± 0.30^a^	24.09 ± 0.56^a^	26.09 ± 0.43^a^
	45 Days	24.08 ± 0.56^b^	24.17 ± 0.47^b^	23.06 ± 0.32^c^	21.01 ± 0.92^d^	23.08 ± 0.81^b^
	90 Days	22.11 ± 0.65^e^	21.17 ± 0.72^f^	20.12 ± 0.61^f^	20.12 ± 0.81^f^	21.12 ± 0.95^d^
Linoleic acid	1st Day	2.79 ± 0.56^a^	2.75 ± 0.72^a^	2.73 ± 0.95^a^	2.79 ± 0.29^a^	2.77 ± 0.91^a^
	45 Days	2.13 ± 0.76^b^	2.43 ± 0.86^b^	2.63 ± 0.92^b^	2.53 ± 0.22^b^	2.59 ± 0.54^b^
	90 Days	1.2 ± 0.73^f^	1.10 ± 0.22^f^	1.79 ± 0.25^c^	1.61 ± 0.24^d^	1.53 ± 0.91^e^
Linolenic acid	1st Day	0.48a ± 0.01^a^	0.46 ± 0.02^a^	0.48 ± 0.06^a^	0.45 ± 0.01^a^	0.50 ± 0.03^a^
	45 Days	0.24 ± 0.02^b^	0.27 ± 0.01^b^	0.25 ± 0.01^b^	0.26 ± 0.02^b^	0.27 ± 0.01^b^
	90 Days	0.15 ± 0.00^c^	0.13 ± 0.01^c^	0.1 ± 0.02^c^	0.18 ± 0.02^c^	0.16 ± 0.03^c^
Arachidic acid	1st Day	2.56 ± 0.15^a^	2.55 ± 0.43^a^	2.51 ± 0.98^a^	2.45 ± 0.32^a^	2.56 ± 0.26^a^
	45 Days	2.51 ± 0.56^a^	2.46 ± 0.32^a^	2.52 ± 0.64^a^	2.46 ± 0.21^a^	2.45 ± 0.16^a^
	90 Days	2.48 ± 0.23^b^	2.47 ± 0.24^b^	2.46 ± 0.76^b^	2.48 ± 0.54^b^	2.41 ± 0.39^b^
Eicosenoic acid	1st Day	0.73 ± 0.24^a^	0.72 ± 0.29^a^	0.75 ± 0.67^a^	0.71 ± 0.37^a^	0.73 ± 0.54^a^
	45 Days	0.52 ± 0.34^b^	0.51 ± 0.65^b^	0.53 ± 0.78^b^	0.50 ± 0.69^b^	0.56 ± 0.45^b^
	90 Days	0.34 ± 0.79^c^	0.32c ± 0.56^c^	0.34 ± 0.69^c^	0.31 ± 0.42^c^	0.35 ± 0.19^c^
C18:1 *t*10–11 (CLA)	1st Day	1.89 ± 0.06^a^	1.88 ± 0.04^a^	1.86 ± 0.02^a^	1.91 ± 0.01^a^	1.89 ± 0.03^a^
	45 Days	1.85 ± 0.02^a^	1.86 ± 0.03^a^	1.82 ± 0.06^a^	1.83 ± 0.06^a^	1.86 ± 0.05^a^
	90 Days	1.71 ± 0.07^b^	1.69 ± 0.01^b^	1.55 ± 0.09^b^	1.59 ± 0.10^b^	1.44 ± 0.02^b^

Values are expressed as mean ± SD of three replicates

^a,b,c,d,e,f^ different short case letters within column showed significant (*P* < 0.05) relationship

M4.9 (Cheese heat stretched at milling pH 4.9), M 4.8 (Cheese heat stretched at milling pH 4.8), M5.0 (Cheese heat stretched at milling pH 5.0), M5.1 (Cheese was heat stretched at milling pH 5.1), M5.2 (Cheese was heat stretched at milling pH 4.9)

### Sensory evaluation

#### Color change in mozzarella cheese

The whiteness of cheese samples decreased significantly (*P* < 0.05) with milling pH and storage as indicated from Table 4.

**Table 4:** Mean and standard error of color, appearance and texture scores of buffalo milk mozzarella cheese at different milling pH during storage

Treatments	Storage Days	Color (Whiteness)	Appearance (Score)	Texture (Score)
M4.9 (Control)	1st	55.23 ± 0.95^a^	8 ± 0.78^a^	7.33 ± 0.61^a^
	45 days	51.23 ± 0.65^b^	9 ± 0.65^b^	6.22 ± 0.34^b^
	90 days	47.23 ± 0.61^c^	6.96 ± 0.61^c^	5.33 ± 0.45^c^
M4.8	1st day	57.06 ± 0.35^a^	7 ± 0.51^a^	6.13 ± 0.34^a^
	45 days	49.06 ± 0.51^b^	8 ± 0.72^b^	5.13 ± 0.23^b^
	90 days	46.06 ± 0.78^c^	6.33 ± 0.91^c^	4.13 ± 0.65^c^
M5.0	1st day	58.2 ± 0.71^a^	5 ± 0.65^a^	8.00 ± 0.34^a^
	45 days	53.2 ± 0.56^b^	6 ± 0.63^b^	7.23 ± 0.57^b^
	90 days	49.2 ± 0.87^c^	4 ± 0.61^c^	6.23 ± 0.98^c^
M5.1	1st day	69.36 ± 0.13^a^	4.23 ± 0.67^a^	9.0 ± 0.56^a^
	45 days	65.36 ± 0.62^b^	5.23 ± 0.62^b^	8.26 ± 0.35^b^
	90 days	62.36 ± 0.6^c^	3.23 ± 0.56^c^	7.26 ± 0.56^c^
M5.2	1st day	74.93 ± 0.97^a^	4.3 ± 0.67^a^	9.00 ± 0.45^a^
	45 days	71.26 ± 0.91^b^	5.3 ± 0.34^b^	8.26 ± 0.02^b^
	90 days	68.26 ± 0.82^c^	3.3 ± 0.78^c^	7.26 ± 0.65^c^

Values are expressed as mean ± SD of three replicates

^a,b,c^ different short case letters within column showed significant (*P* < 0.05) relationship

M4.9 (Cheese heat stretched at milling pH 4.9), M 4.8 (Cheese heat stretched at milling pH 4.8), M5.0 (Cheese heat stretched at milling pH 5.0), M5.1 (Cheese was heat stretched at milling pH 5.1), M5.2 (Cheese was heat stretched at milling pH 4.9)

#### Appearance

Appearance is a quick indication of cheese quality which indicates surface consistency and fusion of cheese shreds after cooking. The results of appearance scores are shown in Table 4. Appearance scores of cheese samples generally increased with decreasing milling pH however appearance scores decreases with storage.

#### Texture

With respect to hardness; cheese showed less compression force with decreasing milling pH Table 4. Maximum force was observed at 5.2 pH while lowest found at 4.8.

## Discussions

The results demonstrated that decreasing milling pH from 5.2–4.9 resulted in decrease in moisture content of cheese. It is because of low pH which tends to unstable the protein network which releases more moisture [27].With reducing milling pH from 5.2 to 4.9 it was seen that starter cultures caused to increase titratable acidity form 0.70 to 1.78 which in turn increase the amount of whey separation [28]. As a result of the changes of the milling pH, all the cheeses experienced a significant loss in protein content. This was detected as a continuous decrease in whey level throughout cheddaring period which is a rich source of protein [29]. In contrast to protein content, fat content of cheese increases with decreasing milling pH. It might be due to more time taken to stretch the cheese which loses the fat contents of cheese [30]. Buffalo mozzarella cheese does not stretch at higher milling pH (from 5.2 to 5.0) which increases fat loses during stretching. Ash contents of cheese decreased with decreasing milling pH. It is due to development of acid that solubilize the micellar calcium phosphate and acting as a vehicle to expel minerals in whey [18]. The level of calcium decreases with decreasing milling pH while there is no clear trend observed for potassium and sodium during change in milling pH. The effect of milling pH on drainage affects calcium as the majority of the non-micellar calcium is lost in the whey. The changing of the pH at milling influences the level of calcium lost in the whey [27]. Storage affects minerals composition non- significantly (*P* > 0.05).

Processing condition (milling pH) has no effect on the fatty acid profile of Mozzarella cheese. During the storage period of 90 days, concentration of unsaturated fatty acids decreased and saturated fatty acids increased on percentage basis. Literature also support the findings of this investigation, amount of unsaturated fatty acids decreased in the long term storage of cheddar cheese [31]. Fatty acid profile of fresh and ripened cheddar cheese is different, also confirmed by the [32]. Concentration of C18:1 *t*10–11in freshly prepared Mozzarella cheese was 1.89%. Milling pH had no effect on the concentration of C18:1 *t*10–11, storage up to 45 days had a non-significant effect, after 90 days of storage of Mozzarella cheese, concentration of C18:1 *t*10–11in was significantly lower than the fresh cheese. Storage duration had a significant effect on concentration of conjugated linoleic acid in milk fat [33]. Cheese samples was wrapped in transparent plastic pouches, which have increased the phot-oxidation of lipids. Moreover, packaging materials used for cheese samples have reduced barriers towards light and oxygen which may result the oxidation of cheese. Alves et al. [34] also find out that Requeija o cremoso cheese get oxidized after exposure to light. It is concluding that cheese may get oxidized during transportation as well as inappropriate storage conditions rather than during modern production system [35].

At high milling pH protein network is less dense that allows more scattering of light which gives whiter color to cheese while as the milling pH decreased, the protein network becomes more compact and dense, the structure becomes more homogeneous that resist scattering of light and whiteness decreases [36]. The same happened with ripening when the casein network becomes more swollen and decreased light scattering consequently decreased whiteness of cheese. At higher milling pH the cheese were with grainy structure and did not fuse and stretch. However, surface rupture decreases as the milling pH decreases till 4.9 milling pH it is due to increase in elongation and strong casein network but as the storage period increases due to biochemical breakdown of protein network cheese appearance scores reduced as the cheese losses its consistency to stretch but fusion increases [37]. Cheese becomes soft as milling pH reduced due to dissociation of casein network in to smaller aggregates. As the pH drops, the casein integrity also lost [38]. This was probably because of the more compact structure of the cheese as lower pH of cheese curd decreases the electrostatic repulsion and strengthens interactions within and between casein aggregates which result in cluster formation and firmer texture [39].

## Conclusion

Complete information about the fatty acid profile of Mozzarella cheese is the concerning matter of consumer’s health. Fatty acid profile of buffalo mozzarella cheese at various milling pH is demonstrated in this study. Buffalo milk cheese is rich with the saturated fatty acid but also exhibit unsaturated fatty acid that has strong health impact to control cardiovascular diseases and reduce the risk of cancer. It is a confusing nutritional concept among consumers about industrial scale produced cheese as it has less flavor and texture qualities as compared to artisanal cheese. In the present study it is concluded that processing technologies minimally influence the fatty acid profile of cheese rather than post manufacturing conditions. The study showed that unsaturated fatty acids of mozzarella cheese are chemically more sensitive as compared to saturated fatty acids and decreases with the storage of mozzarella cheese if it is not properly packed or stored.

## Abbreviations

CLA: Conjugated Linoleic Acids
FA: Fatty acids
MUFA: Monounsaturated fatty acids
PUFA: Polyunsaturated fatty acids
SFA: Saturated fatty acids
